# Follow-up value of serum AFP and aminotransferases in chronic hepatitis B progression

**DOI:** 10.3389/fcimb.2023.1082390

**Published:** 2023-01-25

**Authors:** Mengyao Yu, Lei Huang, Shichang Zhang, Longfeng Jiang, Yuexinzi Jin, Min Gu, Jun Liao, Jiexin Zhang

**Affiliations:** ^1^ Department of Laboratory Medicine, The First Affiliated Hospital with Nanjing Medical University, Nanjing, China; ^2^ Branch of National Clinical Research Center for Laboratory Medicine, Nanjing, China; ^3^ Department of Laboratory Medicine, Nanjing Medical University, Nanjing, China; ^4^ Department of Infectious Diseases, The First Affiliated Hospital with Nanjing Medical University, Nanjing, China; ^5^ School of Science, China Pharmaceutical University, Nanjing, China

**Keywords:** chronic hepatitis B, alpha-fetoprotein, aspartate aminotransferase, acute hepatitis flare, outpatient follow-up

## Abstract

**Introduction:**

Chronic viral hepatitis (CH) is a stage prior to cirrhosis and primary cancer. Standard protocols for CH assessment during the long follow-up period are of great importance for precise treatment and living quality improvement. In this study, we aimed to analyze multiple serum indexes in chronic hepatitis B (CHB)-infected patients and to discuss their combined values in clinical applications.

**Methods:**

Total 503 lines of laboratory data from 2012 to 2021 were extracted from103 CHB patients who were followed-up in our hospital. They were divided into the remission group and the progression group according to their complete clinical information and laboratory data. A series of models of serum indexes were analyzed to illustrate the fluctuation trend of @ach index in a time-dependent manner.

**Results:**

The models revealed that abundant serum alpha-fetoprotein (AFP) in the remission group was characteristically associated with hepatocyte destruction markers aspartate aminotransferase (AST) and alanine aminotransferase and favored a much longer progression-free period (P 0.0001). A model-derived equation consisting of serum AFP and AST values showed a good performance (83% reliability) to distinguish the two groups.

**Discussion:**

This study clearly demonstrates the intrinsic quantitative relationship between serum AFP and liver aminotransferases involving antivirus treatment response. The model-based equation compensates for serum hepatitis B virus DNA detection during outpatient follow-up and it may serve as a useful laboratory tool for CHB progression assessment.

## Introduction

Viral hepatitis is a severe public health problem affecting hundreds of millions of people worldwide. Hepatotropic viruses mainly include hepatitis B virus (HBV), hepatitis C virus (HCV), hepatitis D virus (HDV) and hepatitis E virus, among which HBV, HCV and HDV are the major causes of progressive hepatic fibrosis, liver failure, and hepatocellular carcinoma (HCC) (Buti et al., 2022). China has an over-weighted HBV infection burden, with an estimated 93 million carriers of hepatitis B surface antigen (HBsAg), of which 20~30 million have progressed to chronic viral hepatitis (CVH) (Cui and Jia, 2013; Wang et al., 2014; Liu et al., 2019). Approximately 10~15% of chronic hepatitis type B (CHB) patients are co­infected with HCV and 5% with HDV, which increases morbidity and mortality rates (Seto et al., 2018).

CHB is a chronic necroinflammatory disease of the liver caused by persistent HBV infection (Fung et al., 2022). The different stages of CHB can vary depending on the dynamic interactions between the virus and the liver microenvironment which consists of hepatic parenchymal cells, nonparenchymal cells and local immune cells. Some patients may experience lifelong quiescence, but others will develop more severe complications characterized by fluctuations in HBV DNA and alanine aminotransferase (ALT) levels (Gish et al., 2015). HBV provokes a persistent and chronic immune response to destroy hepatocytes followed by necrosis and fibrosis in situ. With the persistent high viremia as well as the expansion of inflammation and destruction back and forth, ignored or inappropriately treated CHB will progress to cirrhosis or HCC within years (Gines et al., 2021). Therefore, decision making on initiating therapy refers to the virological assessment and the primary goal is to eliminate or significantly suppress HBV replication which is often represented by serum HBV DNA level (Lok et al., 2016; Naggie and Lok, 2021). The first-line therapies worldwide are pegylated interferon alfa, entecavir (ETV), tenofovir disoproxil fumarate, and tenofovir alafenamide (Martin et al., 2022). Nucleotide/nucleoside lamivudine (LAM) was once a commonly used therapy in China because of its established long-term safety and low-cost despite of its comparatively higher risk of the emergence of therapy-resistant mutations (Sarin et al., 2016). In order to achieve a functional cure (HBsAg loss), leading professional associations, such as the American Association for the Study of Liver Diseases, the European Association for the Study of the Liver, and the Asian Pacific Association for the Study of the Liver, agree on the requirement for indefinite long-term or life-long therapy in the vast majority of patient (Block et al., 2021).

The Model for End-stage Liver Disease (MELD) score and Child-Turcotte-Pugh (CTP) score are two systems that are comprehensively used to evaluate the severity of liver cirrhosis and have achieved fruitful clinical results so far. However, there is still a lack of systematic data or evidence related to the establishment of CHB models, especially those that can be used to assess the real-time status of the remaining healthy hepatocytes during outpatient follow-up.

Alpha-fetoprotein (AFP) is derived from embryonic endoderm tissue cells and is a constitutional component of fetal circulation. The serum AFP level gradually decreases after birth to less than 10 ng/mL in adulthood, in which it is synthesized and secreted by liver stem/progenitor cells (LPCs) (Jiang et al., 2022). Overexpressed AFP is well known as an HCC biomarker. Nevertheless, some studies have focused on its indication in liver regeneration (Bloomer et al., 1977; Taketa, 1990; Chen et al., 2017; Jiao et al., 2021). Wang et al. demonstrated that a high serum AFP level (log_10_AFP≥ 2.04) can predict 180-day survival and better outcomes [decreased total bilirubin (TBIL) and international normalized ratio (INR)] in patients with hepatitis B-related acute-on-chronic liver failure (Wang et al., 2018). Their further study revealed that AFP is a candidate marker to be representative of active liver regeneration and it is also an independent prognostic factor in these patients (Wang et al., 2020).

In this study, we retrospectively collected the clinical and laboratory data of patients who had suffered from CHB for years to investigate the relationship of serum AFP and other serum indexes in model establishment and to evaluate their significance in CHB progression or regression.

## Materials and methods

### Patient population

We screened treatment-naïve CHB patients in our hospital and the enrolled CHB patients must had: (1) viremia (serum HBV DNA copy number ≥ 1 × 10^3^ IU/mL) before antiviral drug (ETV or LAM) administration between 2012 to 2019; (2) virological response (serum HBV DNA copy number < 20 IU/mL) after antiviral therapy; (3) continuous administration of the same antiviral drug during outpatient follow-up; and (4) at least one time point of serum AFP, ALT, aspartate aminotransferase (AST), TBIL, direct bilirubin (DBIL), total protein (TP), albumin (ALB), and coagulation detection during viremia and at least one time point after virological response.

The exclusion criteria were as follows: (1) only one time point or no serum AFP, ALT, and AST values; (2) irregular antiviral drug administration; (3) lost to follow-up (including death); and those with (4) acute hepatitis; (5) alcoholic cirrhosis; (6) prior decompensation; (7) metabolic fatty liver; (8) hepatic cancer; (9) autoimmune hepatitis; (10) non-hepatitis B virus infection or HBV-combined hepatitis virus infection; and (11) other diseases, including cardiovascular disease, renal insufficiency, hypersplenism, and metabolic disorders.

### Patient grouping, follow-up, and data collection

Total 103 enrolled CHB patients were classified into two groups: the progression group and the remission group. The progression group contained 52 patients including 18 with CTP grade A/B. After virological response, they had acute hepatitis flare with recurrent viremia (increase of HBV DNA by > 1 × log_10_ IU/mL, n=17) or a re-elevated serum ALT level greater than 2 times the upper limit of normal (> 70 U/L for men and > 50 U/L for women, n=11), or they had persistent liver inflammation and dysfunction with higher levels of multiple serum indexes [e.g., ALT, AST, TBIL, DBIL, and international normalized ratio (INR), n=24] (Vittal and Ghany, 2019). The remission group contained 51 patients including 16 with CTP grade A/B. They showed persistently undetectable serum HBV DNA levels and normal serum indexes levels after virological response.

All follow-up laboratory test results were documented backwards and forwards from the day of initial viremia and were sorted by the detection date of serum indexes. Data collection stopped just before documented therapy switch or disease progressed in the progression group. Total 503 lines of data were acquired from 2012 to 2021 and were respectively analyzed by month intervals between the dates of follow-up test and initial viremia (**Supplementary Figures 1, 2**). This study was approved by the Research Ethics Committee of the First Affiliated Hospital of Nanjing Medical University (2021-SR-570).

### Model establishment and equation generation

Scatter plots (ggplot2 package), quantile-quantile plots (car package), predicted plots of the generalized-additive-model (GAM) (mgcv package), predicted GAMs for serum indexes of log transformation with 90% bootstrap pointwise confidence intervals, and 2D partitions plot of the Quadratic-Discriminant-Analysis (QDA) (klaR package) were all established by R language (Version 4.1.2, R Core Team, Austria) with the original data.

### Trend model selection

By adjusting R^2^ and mean square error (MSE), we performed a stepwise selection and chose a model with best performance in total six nonlinear models (polynomial regression, step function, regression spline, smoothing regression, local regression, and GAM).

### Internal validation

Interval validation was performed by bootstrap sampling. The GAM was fit on the bootstrap dataset for each of the 1000 bootstrap samples. The outcomes on the full dataset were predicted and the R^2^ and MSE from these predictions were calculated. Then, the mean R^2^ and MSE across bootstrap samples as well as standard errors were calculated to obtain the final confidence intervals.

### Classification model selection

Total six classification models were used to respectively predict the qualitative response variable, which were logistic regression, linear discriminant analysis, support vector classifier, support vector machine with linear kernel, support vector machine with radial kernel, and QDA. A model with the lowest test error was chosen for subsequent analysis.

### Variable selection

Best subset selection was used to identify the best model for daily clinical practice. All combinations of the predictors were performed and were compared to fit model. Leave-One-Out-Cross-Validation (LOOCV) was used to calculate the test classification error of each model. A model with the smallest error was selected as the optimal model for subsequent analysis.

### Statistical analysis

Data were analyzed using GraphPad Prism 8.0 (GraphPad, San Diego, CA, USA, https://www.graphpad.com/scientific-software/prism/). Descriptive analyses were conducted using the chi-square test for categorical variables and Student’s t-test for continuous variables. Group differences were examined using unpaired t tests for normally distributed variables or using Mann–Whitney U tests for nonnormally distributed data. The significance level was set at P<0.05.

## Results

### Laboratory values of enrolled patients

**Figure 1:**
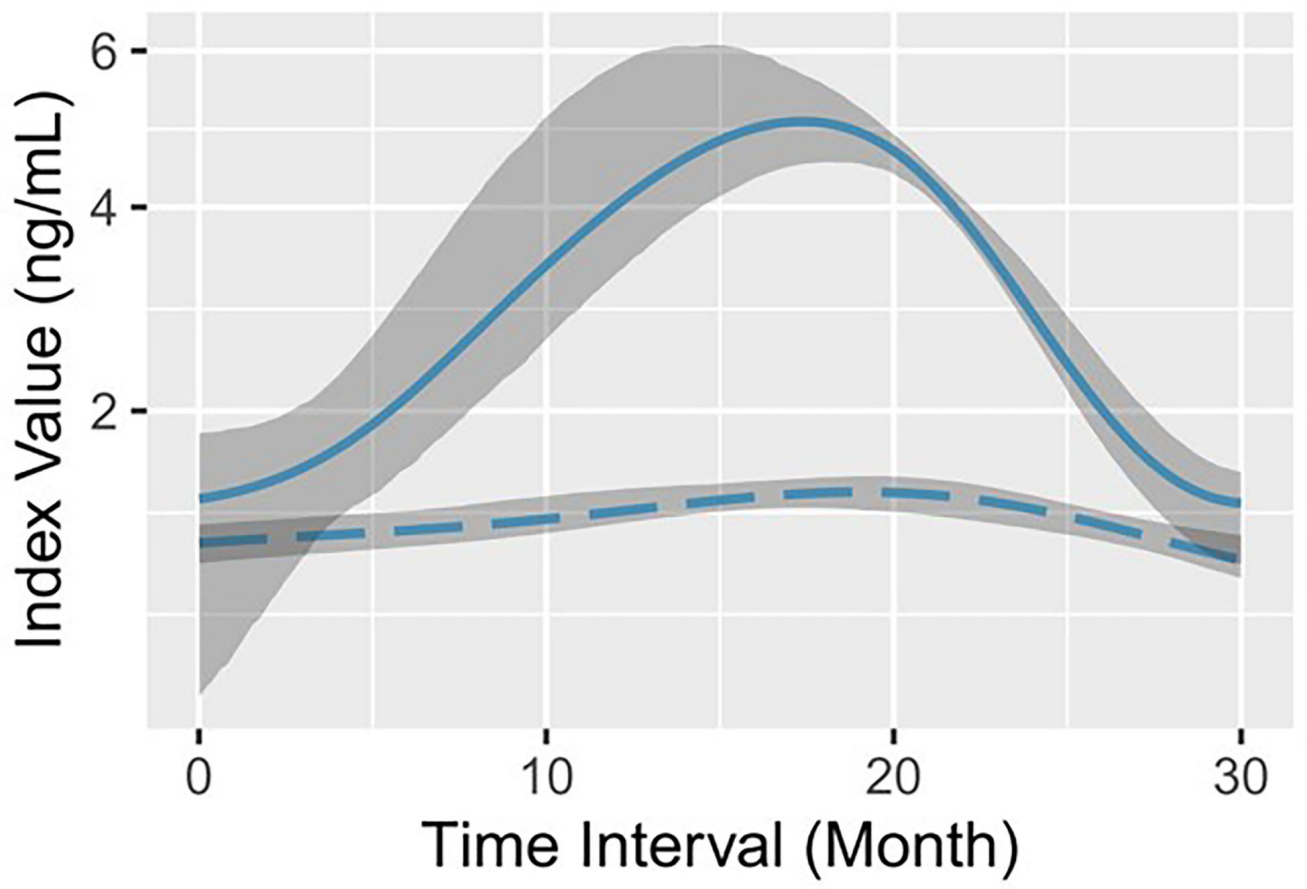
Log-predicted GAMs with 90% bootstrap pointwise confidence intervals for serum AFP. The solid line (representing remission) and the long-dashed line (representing progression) indicate the log-fitted GAM of serum AFP in the remission group and the progression group, respectively. The shadow area corresponds to the 90% bootstrap resampling pointwise confidence interval.

A total of 103 middle-aged CHB patients were enrolled, of which 75.7% were male. According to the medical records after virological response, all patients were divided into the progression group and the remission group (see details in “Patient grouping” in Materials and methods). The progression group contained 234 lines of data from 52 patients and the remission group contained 269 lines of data from 51 patients. During initial viremia, a flare of hepatocyte destruction was indicated in the remission group with statistically higher serum ALT (P<0.0001) and AST (P=0.0002) levels (**Table 1**). In the meantime, the average serum AFP level was significantly higher in the remission group (median: 30.1 ng/mL) than in the progression group (median: 3.1 ng/mL) (P<0.0001; **Table 1**). Then, a GAM was established to observe the serum AFP fluctuation trend in each group based on the follow-up time. We found that the optimal fitting of time width by GAM analysis was 30 months (**Figure 1**). The remission group had a quick increase followed by a rapid decrease to the baseline, whereas the progression group barely exhibited an elevation (**Figure 1**). Moreover, the magnitudes of the two AFP curves were completely different.

**Table 1:** Patients’ information and laboratory test results on the date of initial viremia.

	Remission (n=51)	Progression (n=52)	Statistic	P-Value
Gender^1^: Male N(%)	42(82.4)	36(69.2)	-	-
Age^2^	47.51 ± 13.25	44.79 ± 14.51	t=0.9934	0.3229
ETV/LAM^1^: N(%)/N(%)	42(82.4)/9(17.6)	47(90.4)/5(9.6)	–	–
Serum variables^3^				
AFP (ng/mL)	30.1 [6.0, 167.3]	3.1 [2.1, 4.8]	U = 8926	<0.0001
ALT (U/L)	54.6 [29.9, 130.2]	40.3 [23.4, 79.2]	U = 23896	<0.0001
AST (U/L)	54.0 [36.0, 111.2]	45.7 [32.9, 62.5]	U = 24287	0.0002
TBIL (µmol/L)	29.6 [16.7, 70.2]	19.4 [11.2, 44.1]	U = 22359	<0.0001
DBIL (µmol/L)	13.2 [5.8, 46.3]	7.7 [4.0, 16.7]	U = 20681	<0.0001
TP (g/L)	70.2 [61.6, 77.2]	70.5 [62.8, 75.0]	U = 29496	0.4299
ALB (g/L)	37.5 [32.6, 42.5]	41.1 [32.6, 44.7]	U = 26091	0.0034
HBV DNA copy number (IU/mL)^3^	1130000 [3760, 17100000]	61800 [2095, 24050000]	U = 1087	0.1148
HBeAg status^1^	51(100)	52(100)	–	–

^1^presented as number (percentage); ^2^presented as X ± SD; ^3^presented as median [interquartile range].

### Serum AFP is responsible for progression-free period

**Figure 2:**
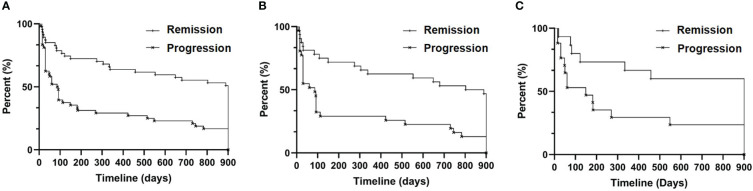
Progression-free survival analysis. **(A)** All patients (n=95, eight patients without documented drug dosage were excluded); **(B)** CHB patients (n=63, six patients without documented drug dosage were excluded); and **(C)** CTP grade A/B patients (n=32, two patients without documented drug names were excluded).

We calculated the time interval between the initial viremia and the subsequent rescue therapy for acute hepatitis flare within 30 months for each patient. The maximum interval was 900 days. The median progression-free period was 900 days in the remission group and 88.5 days in the progression group (HR: 0.5214, 95% CI: 0.3418 to 0.7954, P<0.0001; **Figure 2A**). Next, we divided each group of patients into general CHB and CTP grade A/B to respectively analyze their progression-free period. As shown in **Figure 2B**, the general CHB patients in the remission group had significantly longer progression-free period (846 days) than those in the progression group (86 days; HR: 0.5038, 95% CI: 0.2977 to 0.8525, P=0.001). A similar difference was also confirmed between the two CTP grade A/B groups (900 days vs. 149 days; HR: 0.5484, 95% CI: 0.2689 to 1.118, P=0.0261; **Figure 2C**).

### Composite values of serum ALT, AST and AFP

**Figure 3:**
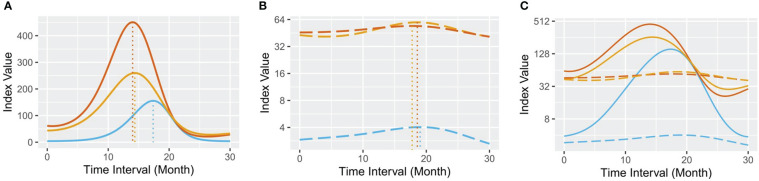
Predicted GAM plots for serum AFP, ALT and AST. **(A)** the remission group; **(B)** the progression group; and **(C)** all samples. In each plot, the blue line represents the GAM of serum AFP, the red line represents the GAM of serum ALT, and the orange line represents the GAM of serum AST. The full lines represent the remission group, and the long-dashed lines represent the progression group.

Next, we combined the hepatocyte markers ALT and AST with AFP to explore whether they interact by GAM analysis. Interestingly, they presented an exquisite coherent relationship in the remission group (**Figure 3A**). The results showed that 1) the wave crest of ALT emerged ahead of that of AST; 2) in response to increased serum ALT and AST concentrations, AFP secretion was upregulated; 3) serum ALT and AST levels were then alleviated; and 4) the serum AFP concentration gradually decreased as liver function was restored. Overall, the dynamic changes in serum ALT, AST and AFP exhibited a nested bell-shaped diagram during the 30-month. By contrast, there was no obvious pattern in the progression group (**Figures 3B, C**). Spearman analysis confirmed the strong correlation between serum AFP and AST (r=0.4787, P<0.0001) or ALT (r=0.4425, P<0.0001) in the remission group (**Supplementary Figure 3**).

Next, we used QDA as the best-fit classification model to distinguish the two group patients. As a result, a total of fifteen classification models were generated (**Table 2**), and one model subset [log(AFP) + log(AST)] had the smallest classification error whose area under curve was 0.815 with sensitivity of 0.9283, specificity of 0.7018, and accuracy of 0.8235 (**Supplementary Figure 4**). Then, we generated an accessible equation (Equation 1) that can classify CHB patients into the remission group (rm) or the progression group (pr) based on serum AFP and AST values. According to the larger output value (Equation 2 into Equation 1 vs. Equation 3 into Equation 1), a result with approximately 83% reliability was expected.


(1) $$\delta_{k}(x)=-\frac{1}{2}{\left(x-\mu_{k}\right)}^{T}\sum_{k}^{-1}(x-\mu_{k})-\frac{1}{2}\log|\sum_{k}|+\log\Pi_{k}$$

(2) $$\begin{array}{l} \mathrm{Remission}\;\mathrm{Group}\;(rm):\\ \mu_{rm\;}=\;[3.523648\;4.247261]\\ \sum_{rm}\;=[\frac{3.9513566\;0.6458968}{0.6458968\;0.8576953}]\\ \Pi_{rm}\;=0.4624746\\ x=\;[\log(AFP)\text{ log}(AST)] \end{array}$$

(3) $$\begin{array}{l} \text{Progression }Group\;(pr):\\ \mu_{pr\;}=\;[1.177712\;3.916471]\\ \sum_{pr}\;=[\frac{0.37123007\;0.04572816}{0.04572816\;0.49682352}]\\ \Pi_{pr}\;=0.5375254\\ x=\;[\log(AFP)\text{ log}(AST)] \end{array}$$

**Table 2:** Subset Selection by LOOCV.

Number	Model.Formula	Classification. Error
1	Category ~ TimeInterval	0.416
2	Category ~ log(AFP)	0.185
3	Category ~ log(AST)	0.412
4	Category ~ log(ALT)	0.422
5	Category ~ TimeInterval + log(AFP)	0.195
6	Category ~ TimeInterval + log(AST)	0.373
7	Category ~ TimeInterval + log(ALT)	0.379
8	Category ~ log(AFP) + log(AST)	0.176
9	Category ~ log(AFP) + log(ALT)	0.185
10	Category ~log(AST) + log(ALT)	0.418
11	Category ~ TimeInterval + log(AFP) + log(AST)	0.197
12	Category ~ TimeInterval + log(AFP) + log(ALT)	0.199
13	Category ~ TimeInterval + log(AST) + log(ALT)	0.361
14	Category ~ log(AFP) + log(AST) + log(ALT)	0.181
15	Category ~TimeInterval + log(AFP) + log(AST) + log(ALT)	0.187

## Discussion

We reviewed studies published in the last twenty years on clinical laboratory indexes that can reflect hepatocyte death or dysfunction. It seems that their optimistic effects on reducing mortality or complication incidence in CVH patients did not meet requirements. Although AFP is of great importance in damage repair during CVH, there is still a lack of documentation on how to use it other than as a tumor exclusion indicator to evaluate the capability of hepatocyte regeneration (Notarpaolo et al., 2017).

### Existing scoring systems

Both the CTP and MELD scores are used to determine ongoing cirrhosis (Su et al., 2019; Jepsen et al., 2020). The CTP score incorporates three common laboratory indicators (prothrombin time, serum albumin, and bilirubin) as well as clinical indicators (ascites, hepatic encephalopathy and nutritional status) (Su et al., 2019). Bias may arise due to subjective reasoning among clinicians and the therapeutic impact on exogenous serum albumin (Kamath et al., 2001). The MELD score is based on three variables: serum creatinine (CREA), TBIL and the INR. While CREA indicates muscle cell metabolism and is a sensitive index for renal function evaluations (Mindikoglu and Pappas, 2018), bilirubin is the main metabolite of iron porphyrin compounds in senescent red blood cells and reflects the excretion function of the liver (Sullivan and Rockey, 2017). Impeded hepatic cell function also reduces prothrombin production, leading to an increase in the INR (Garg et al., 2012). Other non-disease factors, an insufficient blood volume and excessive diuretic usage contribute to the cumulative error in the final scores. Therefore, all of these indexes are indirect evidence of hepatocyte death, and both of the scoring systems ignore the transition process of the remaining cells (such as regenerating cells) during the lifelong course of CVH.

### Serum AFP’s role in CHB progression assessment

Based on a comprehensive analysis of the medical records and test results of 103 patients, we found that the remission group had abundant serum AFP levels (**Figure 1**) and distinguishable longer progression-free period (**Figure 2**). For a long time, AFP was the most widely used serum marker for the diagnosis of HCC, and its increase indicates a high probability of HCC (the diagnostic criterion is > 20 ng/mL) (Trevisani et al., 2001). Nevertheless, the serum AFP concentration here showed a bell-shaped curve rather than a curve indicating a consistently high level. Our data also indicated that serum AFP has a strong concomitant response to hepatocyte death, characterized by a lagging fluctuation after serum ALT/AST changes in the remission group (**Figure 3A**). This is fundamentally different from that observed in HCC.

LPCs are necessary for liver regeneration. When under attack (such as a virus, drug, or alcohol), LPCs re-express AFP for tissue self-repair and proliferation (Peterson et al., 2011; Kakisaka et al., 2015). Frank’s team measured serum AFP levels on admission (median: 8.1 ng/mL) and on day 3 (median: 17.6 ng/mL) in 162 patients suffering from acute liver failure, and the result showed that AFP upregulation within day 3 may indicate a better prognosis (Schiodt et al., 2006). Schmidt et al. obtained serial serum AFP values at peak in 239 patients with severe acetaminophen-induced liver injury and found that the serum AFP levels in the survivors were significantly higher (from 4.2 ± 3.1 ng/mL to 68.6 ± 77.9 ng/mL) than those in the deceased starting on the day of peak ALT (Schmidt and Dalhoff, 2005). They proposed a combined analysis of AFP and the INR to provide additional prognostic information. From our point of view, a self-limited fluctuation of serum AFP concentration (possibly caused by viral replication and hepatocyte destruction) indicates a longer progression-free period in CHB patients.

### Future practice in laboratory and clinic

Both episodic hepatitis flares and intermittent ALT elevations may occur and repeat during anti-HBV therapy, which more frequently leads to the development of cirrhosis, hepatic decompensation, failure, or even death (Liaw et al., 1988; Chang and Liaw, 2014). Based on this, all recruited patients in this study were divided into two groups. We simultaneously analyzed two more serum indexes, AST and AFP, in CHB patients with adequate liver reserve and highlighted the specific fluctuation pattern of each index in a time-dependent manner before and after effective antiviral therapy. We found that three serum indexes presented an exquisite a nested bell-shaped diagram in the remission group (**Figure 3A**). Jeng et al., reported a phenomenon, called “host-dominating flare” or “beneficial flare”, of greater AFP and ALT fluctuation on HBV DNA fall and rapid HBsAg decline (Jeng et al., 2016; Liaw, 2021). Unlike indexes used in CTP or MELD score system, serum AST and ALT elevation directly reflects aggravated hepatocyte destruction. The hepatocyte regeneration marker AFP whose serum concentration is more critical regarding prognosis was also integrated in our models. The model-derived equation (*Equ 1*) quantifies the relationship between serum AFP and AST and it is convenient for laboratory application. The output of the equation indicates the real-time status of hepatocytolysis and regeneration and help to assess CHB progression in a cost-effectiveness way along with serum HBV DNA measurement during outpatient follow-up. Studies suggested that host-dominating flare is not associated antiviral therapy per se (Chang and Liaw, 2022). Therefore, in-depth research of patients in the remission group may identify the trigger and involved immune responses. It has been proven that fibrosis and early cirrhosis are reversible (Bedossa, 2015). It is also important to recognize patients in the progression group by *Equ 1* and to apply other intervention protocols in a timely manner to improve progression-free period.

### Limitations

Hepatitis B e antigen (HBeAg)-negative CHB patients were not included in this study. They are characterized by impaired HBeAg expression, lower serum HBV DNA levels, more frequent hepatitis flares, and progressive liver damage (Marcellin et al., 2004). Antiviral treatment strategy is primarily urged according to the recommended cut-offs of indications (serum HBV DNA and ALT) for HBeAg-negative CHB, which is quite different from that for HBeAg-positive CHB (Sarin et al., 2016). Since ETV was reported to better improve virological response and liver inflammation than LAM in treatment-naïve HBeAg-negative CHB patients after a 48-week course of treatment (Lai et al., 2006), it is interesting to explore the change patterns of serum indexes in these patients during nucleos(t)ide analogues therapy.

Serum index detection is more acceptable than other invasive examinations for CHB patients during the long course of treatment (usually a lifetime). By establishing connections among the virus, patients and clinicians, this equation provides alternative access to follow-up management from which CHB patients will benefit the most.

## Data availability statement

The raw data supporting the conclusions of this article will be made available by the authors, without undue reservation.

## Ethics statement

The studies involving human participants were reviewed and approved by the Research Ethics Committee of the First Affiliated Hospital with Nanjing Medical University. The patients/participants provided their written informed consent to participate in this study.

## Author contributions

JZ concepted and designed the research. LH, YJ, and MG collected, analyzed, or interpreted the data. JL performed statistical analysis. MY wrote the manuscript. SZ and LJ provided critical revision of the manuscript for important intellectual content. All authors contributed to the article and approved the submitted version.
