# Supplementary material for: Follow-up value of serum AFP and aminotransferases in chronic hepatitis B progression
Source: Front Cell Infect Microbiol. 2023 Jan 25;13:1082390. doi: 10.3389/fcimb.2023.1082390 (PMC9905438; doi:10.3389/fcimb.2023.1082390)
Supplement: Supplementary file 1 [file DataSheet_1.doc]

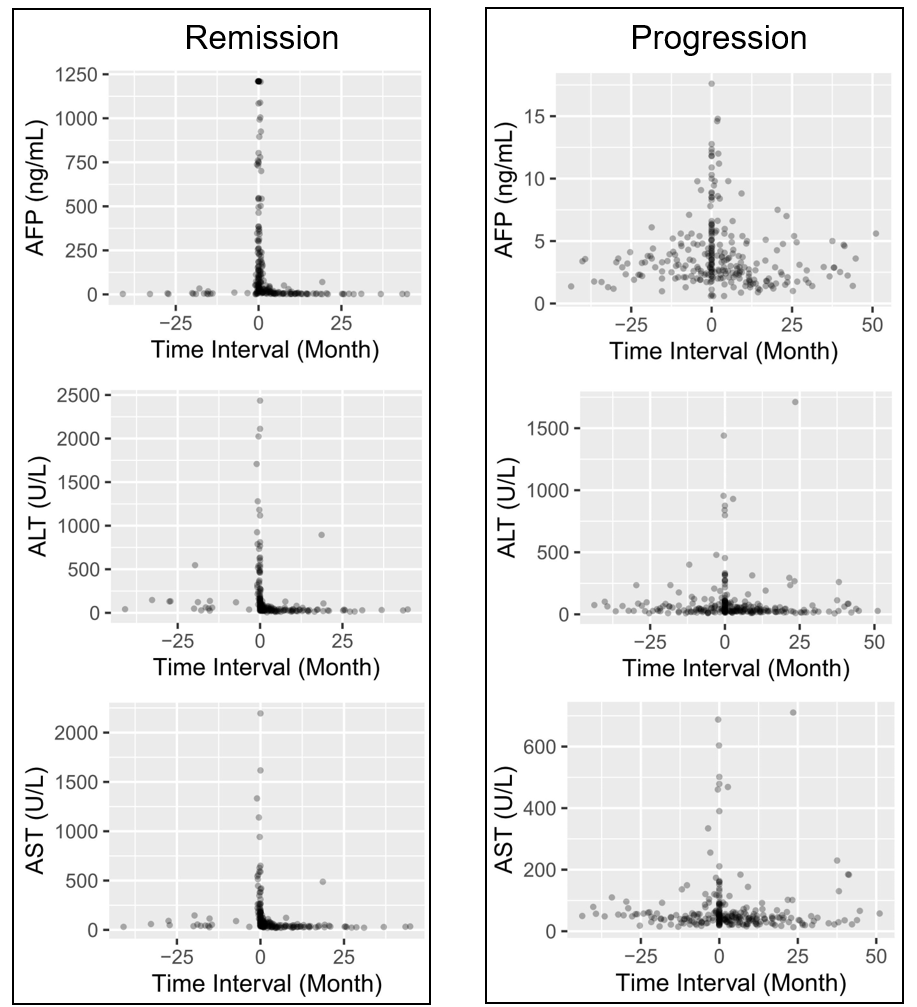


**Supplementary Figure 1.** Scatter plots of the time interval versus three indexes in the remission group and the progression group.


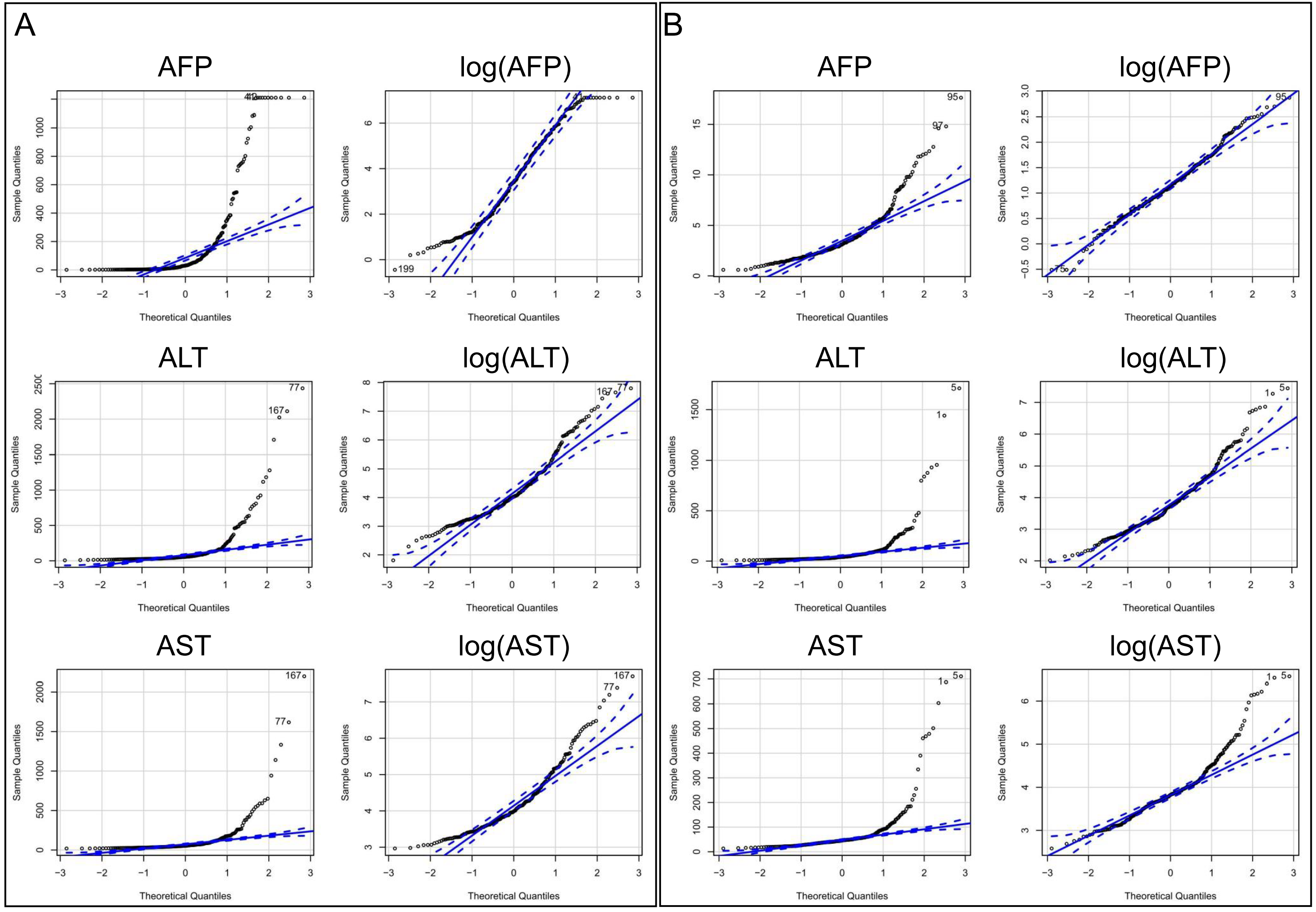


**Supplementary Figure 2.** Quantile-quantile plots of three indexes and their log transformations in the remission group (**A**) or the progression group (**B**). Plots represent the empirical quantiles of the indexes against the theoretical quantiles of comparison distributions. The blue full lines represent perfect quantitle matching, and the blue-dashed lines represent the 95% confidence intervals.


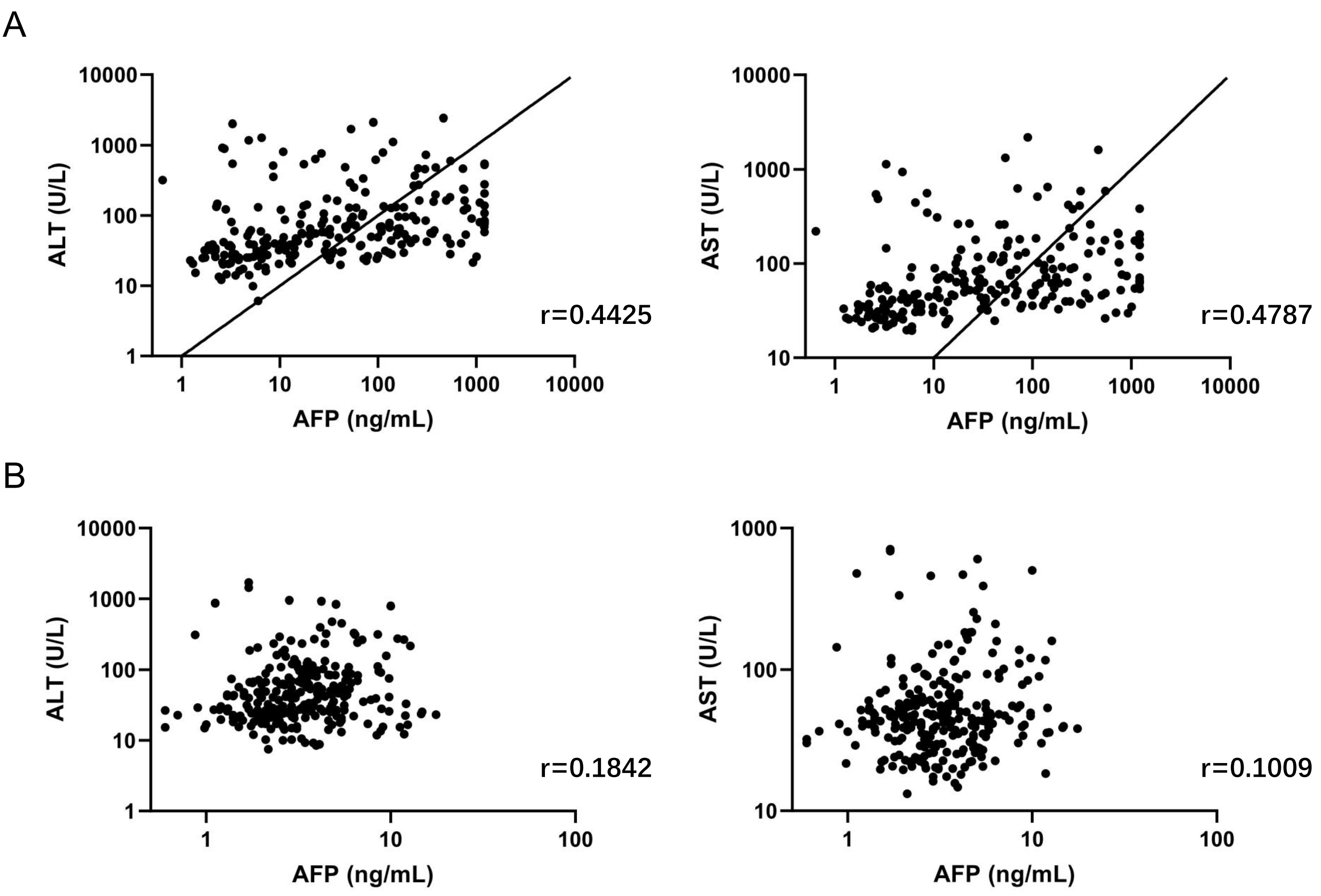


**Supplementary Figure 3.** Spearman analysis results of serum indexes in the remission group (**A**) and the progression group (**B**).


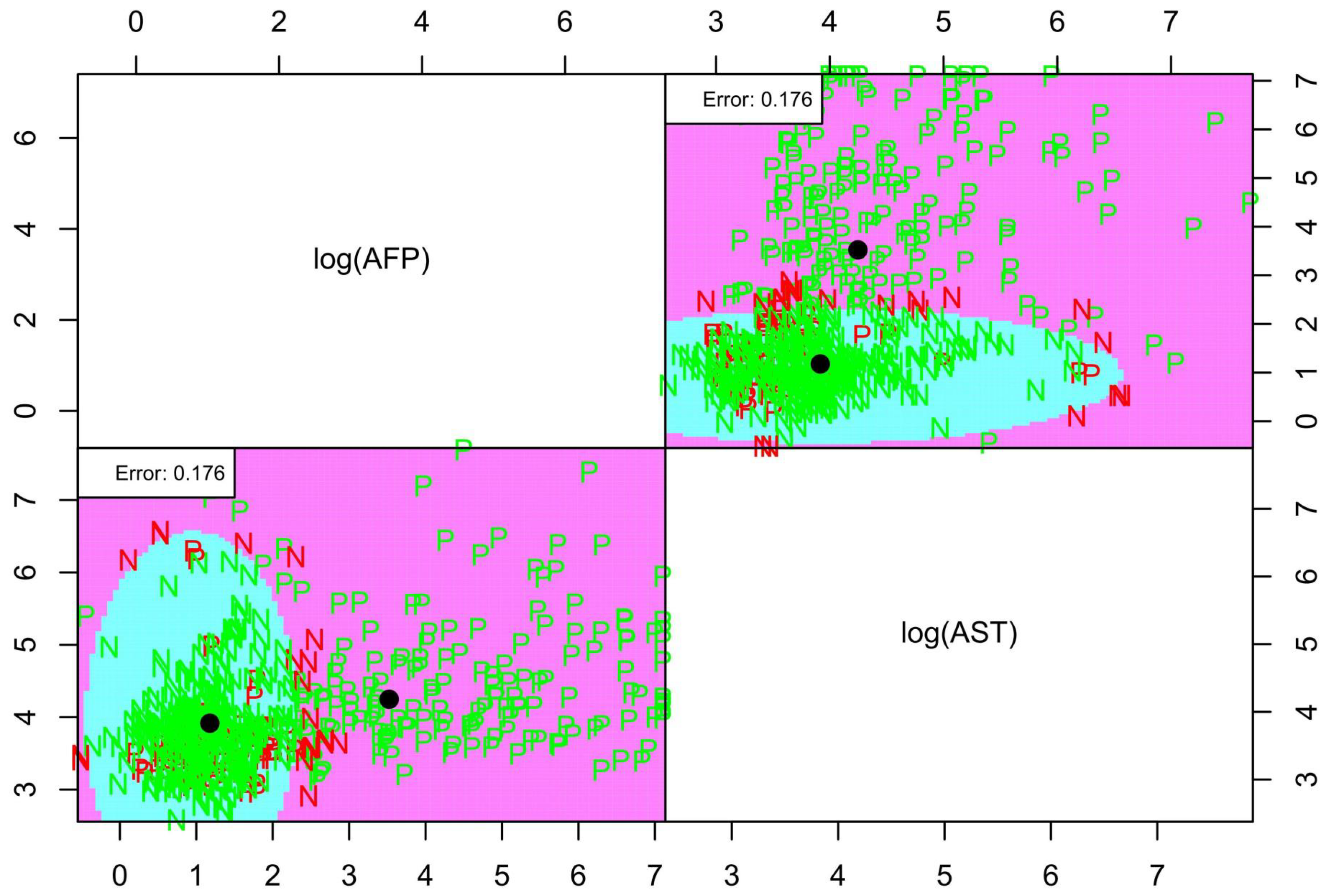


**Supplementary Figure 4.** A 2D partition plot of the QDA. This plot displays the classification of observations based on the QDA of the pair of log(AFP) and log(AST). Each ‘P’ in the plots represents the observations in the remission group, and ‘N’ represents the observations in the progression group. The green observations indicate that the green observation is correctly classified by the QDA, and the red observations indicate that the observation is incorrectly classified by the QDA. Colored regions differentiate each classification area, and the classification errors are given.
